# 736 Intravenous Immunoglobulin in Stevens-Johnson Syndrome & Toxic Epidermal Necrolysis: Experience from a Tertiary Care Center

**DOI:** 10.1093/jbcr/irac012.289

**Published:** 2022-03-23

**Authors:** Manal Hassan, Bao Vincent Ho, Dhaval Bhavsar, Richard Korentager

**Affiliations:** University of Kansas Medical Center, Kansas City, Kansas; University of Kansas School of Medicine, Kansas City, Kansas; University of Kansas Health System, Kansas City, Kansas; University of Kansas Medical Center, Kansas City, Kansas

## Abstract

**Introduction:**

Stevens-Johnson syndrome and toxic epidermal necrolysis (SJS/TEN) are rare severe cutaneous adverse reactions associated with high morbidity and mortality, however, there lacks an established treatment protocol. Treatment with intravenous immunoglobulin (IVIg) has demonstrated mixed success rates in improving mortality. It has been suggested that early intervention with IVIg in SJS/TEN patients may lead to a reduction in observed mortality rate when compared to the predicted rate. We present 24 patients with SJS/TEN treated in our burn unit with IVIg.

**Methods:**

We conducted a retrospective analysis of patients who were hospitalized with the diagnosis of SJS/TEN in a specialized burn center over the years 2011-2020. Data regarding clinical factors, causative agent(s), disease severity, treatment received, and outcome were collected on chart review. SCORTEN and ABCD-10 prognostic scores were calculated for each patient at the time of admission. All patients were started on IVIg at the recommendation of dermatology. A standardized mortality ratio was obtained to compare the actual number of deaths to the predicted number based on SCORTEN and ABCD-10 formulas.

**Results:**

A total of 24 patients were identified with a mean age of 49.8 ± 18.1 years. Most of the patients, i.e., 18 out of 24 had TEN, and 6 patients had SJS/TEN overlap with an overall average initial BSA involvement of 42.7% ± 25.3. Among the suspected drugs, sulfonamide antibiotics (41.7%) was the major predicted culprit. All patients were started on IVIg, 3 of which were treated in combination with corticosteroids. Most of our patients (23/24) received IVIg within 2 days of admission, but on average 11 ± 27 days (range: 2-135) after symptom onset. Many patients (10/24) experienced complications during hospital admission, such as: acute respiratory distress syndrome (8/24), sepsis (6/24), and anemia (2/24). There was no statistically significant difference in the overall observed mortality of 4 patients (16.7%) and the predicted overall mortality of 6.4 patients (26.7%) by the SCORTEN formula [standardized mortality ratio = 0.63; 95% confidence intervals, 0.17-1.61; *P* = 0.48], and the predicted overall mortality of 3.6 patients (15%) by the ABCD-10 formula [standardized mortality ratio = 1.12; 95% confidence intervals, 0.30-2.86; *P* = 0.96].

**Conclusions:**

The findings of the present study do not support the clinical benefits of IVIg for SJS/TEN overlap and TEN patients.

